# Eligibility of real-life patients with COPD for inclusion in RCTs: a commentary

**DOI:** 10.1186/s12931-016-0494-5

**Published:** 2017-01-05

**Authors:** Salvatore Battaglia, Nicola Scichilone

**Affiliations:** DIBIMIS, University of Palermo, Palermo, Italy

**Keywords:** Clinical trials, COPD research, Clinical practice

## Abstract

Randomized clinical trials (RCTs) are performed to provide evidence to support treatment decisions. Based on the nature of those studies and the need to avoid confounding factors, it has been argued that the population selected in RCTs only partially represents the *real-life* population. This assumption casts doubts on the applicability of the results provided by RCTs in the management of individuals with an established diagnosis of COPD, and advocates the need for complementary studies with a pragmatic design. Herein, we comment on the recent article published by Halpin and colleagues on the Journal [Halpin et al, Respir Res 17:120, 2016], in which higher rates of inclusions in RCTs for COPD are found compared to previous observations. By analyzing the design of the studies and the end results, we conclude that the accumulating evidence contribute to shed lights on how representative is the outpatient population of real life settings.

## Background

We read with great interest the article by Halpin and coworkers that aimed at determining the proportion of the general UK patient population with COPD that would be eligible for inclusion in randomized clinical trials (RCTs) testing inhaled long-acting bronchodilators [1]. Current knowledge on the effects of inhaled treatment on the airways of COPD may have been hampered in the past by publication bias towards high internal validity, not sufficiently making the same efforts to test the external validity of clinical trials.

## Main text

In the current study [1], the Authors reported a recruitment rate of inclusion of 23%, arguing against previous work from our [2] and other groups [3–6], which showed lower rates of inclusion. The Authors suggested that the study by Scichilone et al [2] and by Walker et al. [6] could have included a different, and less representative, subset of patients suffering from COPD. We acknowledge that our conclusions of lower rates of inclusion in RCTs were made on a much lower number of patients; however, we respectfully disagree with their assumption that recruiting patients with a more severe disease (from hospital clinics) is a study limitation that raises concerns about the generalisability of the findings. More severe patients deserve more aggressive treatment and this could theoretically lead to higher risk of side effects. Therefore, RCTs should take into account this cluster of patients in order to achieve higher generalisability.

Moreover, Halpin and coworkers concluded that RCTs participants are generally more representative of outpatients than previously believed. The recruitment rate for inclusion of 23% by Halpin’s study is actually very similar to our 17%. This is especially true for the subgroups of RCTs carried out in initial and final five-year periods (years 1999–2003 and 2009–2013) that showed an inclusion rate of 16% and 18%, respectively. The median eligibility rate falls to 13% in the subgroup of RCTs on beta-adrenergics and anticholinergics (LABA/LAMA) combination treatment. This is worrying since novel inhaled combinations of two long-acting bronchodilators are gaining increasing evidence in the pharmacological treatment of COPD [7].

Another aspect that must be highlighted is the possibility that entire categories of patients could be excluded from RCTs: this seems the case of atrial fibrillation. Indeed, the current GOLD recommendations [8] recognize atrial fibrillation as the most frequent cardiac arrhythmia in patients with COPD, while admitting that “*there are no good data on the use of COPD medication in patients with atrial fibrillation and these patients have often been excluded from clinical trials*”. In this contest, the probability that a patient with COPD and concomitant atrial fibrillation is treated without any scientific evidence or safety data is high. Similar observations have been produced in chronic diseases other than COPD [9, 10]. Although under some conditions the eligibility rate could be as high as 58%, overall almost three out of four patients from real life settings are not eligible for RCTs and this should, in our opinion, limit the RCTs generalisability.

Is therefore the ideal RCT the one that allows to include a proportion of “real life” patients close to 100%? It is a possibility. However, an alternative exists: in the era of personalized medicine, RCTs should aim at targeting a very small and well-described proportion of patients, with the totality of patients achieved by pooling results from several RTCs. This is the opposite of the “one-fits-all” approach.

## Conclusions

The current study has the merit of challenging the scientific community on what is (should be) the acceptable eligibility rate that RCTs should achieve in order to be generalizable to real-life COPD populations. We believe that our observations may actually complement those of Halpin and colleagues [1], and pragmatic observational studies or *ad hoc* designed trials should be encouraged to test the effectiveness of inhaled treatment to fill the gap between RCTs patients and every-day patients. Studies such as that by Halpin and coworkers will certainly help to move towards this direction.

## Abbreviations

COPD: Chronic obstructive pulmonary disease
LABA: Long acting beta-adrenergic agonist
LAMA: Long acting muscarinic antagonist
RCT: Randomized clinical trial
UK: United Kingdom

## References

[1] Halpin DMG, Kerkhof M, Soriano JB, Mikkelsen H, Price DB (2016). Eligibility of real-life patients with COPD for inclusion in trials of inhaled long-acting bronchodilator therapy. Respir Res.

[2] Scichilone N, Basile M, Battaglia S, Bellia V (2014). What proportion of chronic obstructive pulmonary disease outpatients is eligible for inclusion in randomized clinical trials?. Respiration.

[3] Herland K, Akselsen JP, Skjonsberg OH, Bjermer L (2005). How representative are clinical study patients with asthma or COPD for a larger “real life” population of patients with obstructive lung disease?. Respir Med.

[4] Travers J, Marsh S, Caldwell B, Williams M, Aldington S, Weatherall M, Shirtcliffe P, Beasley R (2007). External validity of randomized controlled trials in COPD. Respir Med.

[5] Kruis AL, Stallberg B, Jones RC, Tsiligianni IG, Lisspers K, van der Molen T, Kocks JW, Chavannes NH (2014). Primary care COPD patients compared with large pharmaceutically-sponsored COPD studies: an UNLOCK validation study. PLoS One.

[6] Walker S, Fingleton J, Weatherall M, Beasley R (2013). Limited generalisability of UPLIFT findings to clinical practice. Thorax.

[7] Singh D (2015). New combination bronchodilators for chronic obstructive pulmonary disease: current evidence and future perspectives. Br J Clin Pharmacol.

[8] Global Initiative for Chronic Obstructive Lung Disease (GOLD). Global Strategy for the Diagnosis, Management and Prevention of COPD - Update 2016. 2016. Available from: http://www.goldcopd.org/. Accessed 15 Oct 2016.

[9] Battaglia S, Basile M, Spatafora M, Scichilone N (2015). Are asthmatics enrolled in randomized trials representative of real-life outpatients?. Respiration.

[10] Rothwell PM (2005). External validity of randomised controlled trials: “to whom do the results of this trial apply?”. Lancet.

